# Association Between Anatomical Nasal Obstruction and Maxillary Constriction: Study on Postero-Anterior Cephalograms

**DOI:** 10.3390/children13050673

**Published:** 2026-05-13

**Authors:** Fabrizia d’Apuzzo, Ludovica Nucci, Sara Castaldi, Mariagiovanna Ferraioli, Mario Fordellone, Letizia Perillo, Valmy Pangrazio-Kulbersh

**Affiliations:** 1 Multidisciplinary Department of Medical-Surgical and Dental Specialties, University of Campania Luigi Vanvitelli, 80138 Naples, Italy; fabrizia.dapuzzo@unicampania.it (F.d.); letizia.perillo@unicampania.it (L.P.); 2 Department of Mental and Physical Health and Preventive Medicine, University of Campania Luigi Vanvitelli, 80138 Napoli, Italy; ludovica.nucci@unicampania.it (L.N.); mario.fordellone@unicampania.it (M.F.); 3 Private Practitioner, 80129 Naples, Italy; castaldisara13@gmail.com; 4 Department of Precision Medicine, University of Campania Luigi Vanvitelli, 80138 Napoli, Italy; 5 Orthognathic Surgery and Early Treatment Clinics, University of Detroit Mercy Graduate Orthodontic Program, Detroit, MI 48221, USA; vkulbersh@aol.com

**Keywords:** postero-anterior cephalogram, diagnosis, maxillary constriction, nasal obstruction, turbinate hypertrophy, nasal septal deviation

## Abstract

**Highlights:**

**What are the main findings?**

- Anatomical nasal obstruction due to turbinate hypertrophy and/or nasal septal deviation could be easily detected on postero-anterior cephalometry.
- Neither the association between maxillary constriction and turbinate hypertrophy nor that with nasal septal deviation reached statistical significance.

**What are the implications of the main findings?**

- Nasal septal deviation may play a minor role in the development of maxillary transverse deficiency.
- Further studies using three-dimensional imaging and larger samples are needed to confirm these findings.

**Abstract:**

**Objectives:** To investigate the possible association between nasal obstruction and maxillary constriction using postero-anterior (PA) cephalograms in nontreated adolescents. **Materials and Methods:** This retrospective observational study was performed by analyzing the initial PA of 92 subjects in permanent dentition (forty-nine males and forty-three females) who presented without maxillary constriction (control group) and with maxillary transverse deficiency (experimental sample), ages between 12 and 17 years. Patients with any previous orthodontic or surgical treatment of the craniofacial complex were excluded from the study. Linear and angular measurements were taken on PA cephalograms to assess nasal obstruction caused by turbinate hypertrophy and/or the nasal septal deviation. Interval estimation of the prevalence of different variables within gender groups was calculated using the Clopper–Pearson method with a 95% confidence level. The possible associations between maxillary constriction and the presence of nasal anatomical findings were assessed using odds ratios with corresponding 95% confidence intervals. **Results:** More females than males showed maxillary constriction, but the difference was not significant. Neither the association between maxillary constriction and turbinate hypertrophy nor that with nasal septal deviation reached statistical significance. **Conclusions**: The hypothesis was rejected. A deviated nasal septum could be slightly associated with a maxillary constriction. However, these results should be taken with caution due to the bi-dimensionality of the measurements on PA. In contrast, three-dimensional evaluations in a wider sample could provide further outcomes to be discussed.

## 1. Introduction

Nasal breathing plays a critically important role in the development of the dentofacial complex [1]. Consequently, the evaluation and assessment of potential nasal cavity obstruction or impaired respiratory function, possibly induced by a wide range of anatomical, physiological, and pathophysiological factors, is imperative [2]. The nose, the most cranial element of the upper respiratory tract, is responsible for warming, humidifying inhaled air, and filtering it, thereby also exerting a local defense action [3].

The nose can be divided for explanatory purposes into two regions: an external nose and an internal chamber, the nasal cavity [2,3,4]. Each half of the nasal cavity possesses a vestibule, a roof, a floor, and two walls: a lateral and a medial wall. The medial wall of each nasal cavity is formed by the nasal septum, a laminar structure composed anteriorly of a cartilaginous portion and posteriorly of a bony portion, formed by the perpendicular plate of the ethmoid and the vomer. The lateral walls support three protuberances: the inferior, middle, and superior turbinates; each of these structures demarcates a distinct meatus [5,6].

Nasal obstruction is a common occurrence, reported as the sensation of reduced air passage through the nose [7]. The condition can be categorized as chronic or intermittent, with an estimated prevalence of 20–30% across the global population [8].

**Figure 1:**
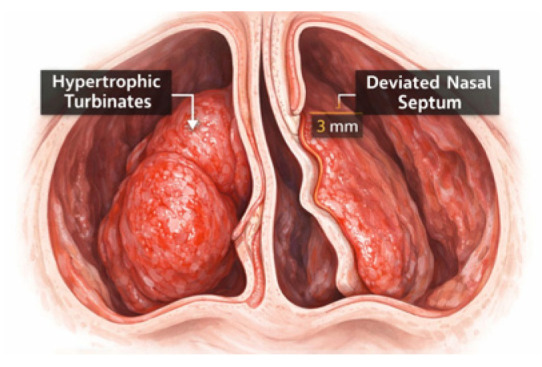
Anatomical nasal obstruction with hypertrophic turbinates and deviated nasal septum.

Causes of nasal obstruction may include anatomical abnormalities, such as nasal septal deviation, which is a condition characterized by displacement of the septum from the midline. Septal deviation is considered the most common cause of nasal obstruction and is estimated to be prevalent in 1/3 of the population [9]. Adenoid hypertrophy, having a reported prevalence of 34.6%, is another anatomical cause that should be considered. The hypertrophy of inferior turbinates can also result in a reduction in nasal valve cross-sectional area through three distinct anatomical changes: bony hypertrophy, soft tissue hypertrophy, and mixed hypertrophy (Figure 1). Following Moss’s theory of functional matrices, physiological nasal breathing allows normal craniofacial and dentofacial growth and development [10,11].

Oral breathing in young patients, which can result from nasal obstruction, was associated with the development of malocclusions during growth, often with skeletal openbite and crossbite, and with a vertical facial growth pattern. This condition is frequently referred to as ‘long face syndrome’ or ‘adenoid facies’ and is marked by an increase in total anterior facial height, a vertical growth pattern, orofacial muscle hypotonia, and lip incompetence. Subjects who are mouth breathers usually have a reduction in palatal arch width, a posterior crossbite, and are associated with Class II dentoskeletal malocclusions [12].

Identification of nasal obstruction and oral breathing pattern is therefore of relevant importance in planning an interceptive treatment approach for young subjects before the end of growth. Further justification for the validity and reliability of the measurement methods for turbinate hypertrophy would strengthen the findings. In a relevant study by Pangrazio-Kulbersh et al. [13], the effect of palatal expansion in mixed dentition subjects treated with different types of maxillary expanders was evaluated, and the dentoskeletal effects at the level of the naso-maxillary complex were assessed by considering, specifically, the soft tissue dimensions of the nasal cavities and maxillary sinuses on three-dimensional images obtained from pre- and post-treatment cone-beam computed tomography (CBCT). However, in children and adolescents, it is preferable to avoid requiring three-dimensional diagnostic examinations such as CBCT based on the ALARA (As Low As Reasonably Achievable) principle, a radiation protection concept applied to limit exposure to ionizing radiation.

Hence, for more correct diagnostic and therapeutic planning in subjects undergoing initial orthodontic screening, it is desirable to request two-dimensional radiographic examinations that equally allow proper assessment of anatomical structures, such as the bidimensional postero-anterior (PA) radiography of the head.

Given the premises, the objective of this investigation was to test the hypothesis that, in young patients with permanent dentition, the presence of nasal obstruction secondary to turbinate hypertrophy and/or nasal septal deviation is significantly associated with decreased transverse maxillary dimensions, as measured on initial posteroanterior (PA) cephalometric radiographs, compared to individuals without evidence of nasal obstruction.

## 2. Materials and Methods

This retrospective observational study was carried out by analyzing the initial records of patients treated at the Program of Orthodontics of the University of Campania *Luigi Vanvitelli* in Naples. The protocol has been evaluated by the Institutional Ethics Committee Campania 2 with Prot. N° 0021814/i, and the research followed the Declaration of Helsinki principles and STROBE guidelines, and all patients’ parents provided their informed consent for the use of personal data.

The collection of PA cephalograms was conducted from July 2024 to February 2025, following the selection criteria: full permanent dentition (except third molars); age below 18 years; high-quality PA x-ray; signed consent by the minor’s parents/tutors to personal data use for research purposes.

**Figure 2:**
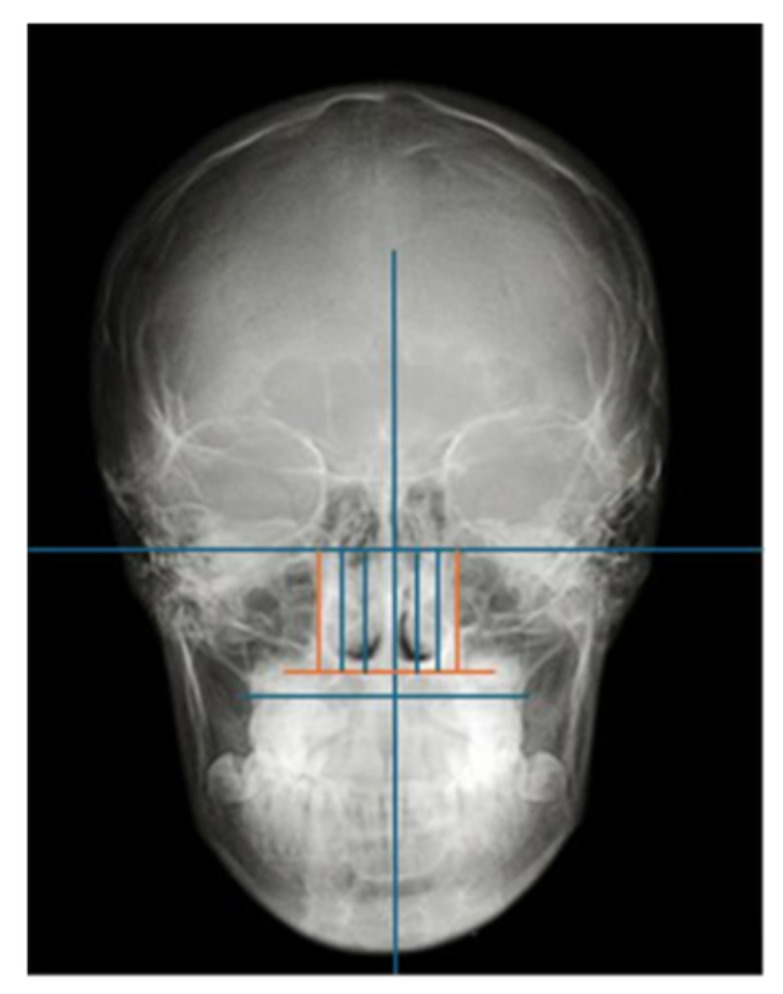
Drawing of the mid-sagittal plane, Frankfurt horizontal plane (PFH), horizontal plane passing through the caudal and lateral ends of the pyriform opening, perpendicular to the mid-sagittal plane and parallel to the PFH, and vertical lines perpendicular to the PFH dividing the right and left nasal cavity into two halves, then divided into thirds.

**Figure 3:**
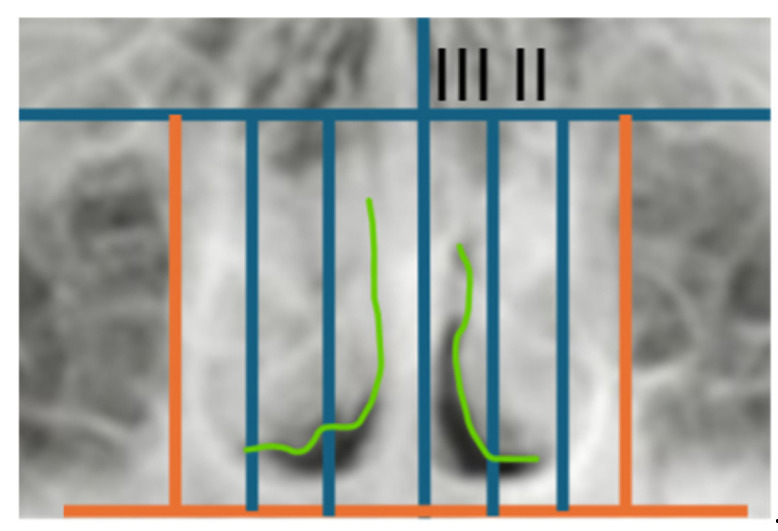
Bidimensional extension of the turbinates highlighted as radiopacity.

**Figure 4:**
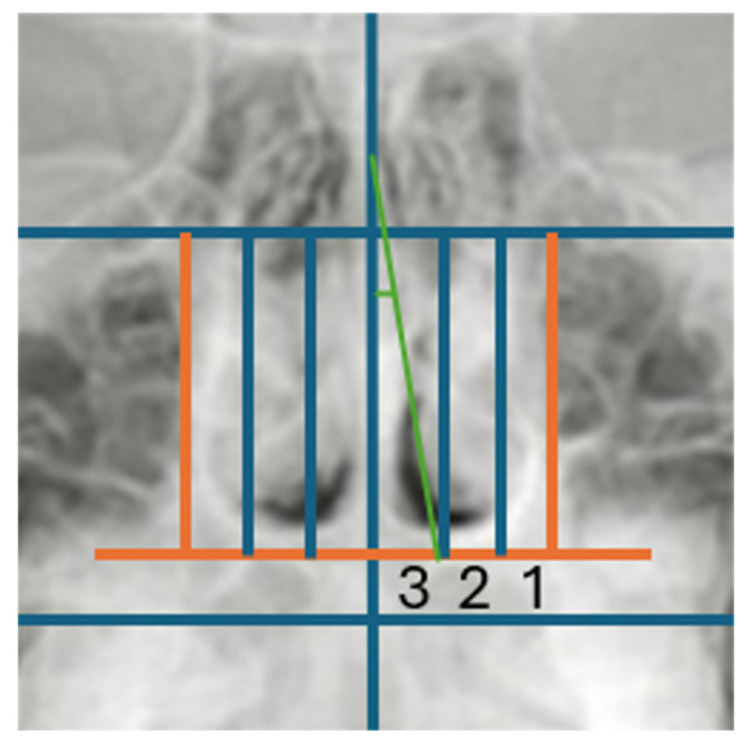
The construction of the angle created between the mid-sagittal plane and the point of convexity of the deviated nasal septum.

The total study sample was separated into two distinct groups after bi-dimensional cephalometric assessment of transversal skeletal values: the first group consisted of individuals without maxillary constriction, which served as the control group, and the second group consisted of individuals with maxillary constriction, which was designated as the experimental group. Specifically, the maxillo-mandibular differential was measured on the PA cephalometry to determine the maxillary constriction according to the patient’s age [14,15]. Further data recording was performed using measurements of the sagittal and vertical diameters of the nasal cavities, turbinates, and nasal septum on a PA cephalometry (Figure 2) evaluated as follows:

(1) mid-sagittal plane constructed from the Crista galli to the Anterior Nasal Spine (ANS) perpendicular to the Frankfurt Horizontal Plane (PFH);
(2) vertical plane tangent to the widest portion of the nasal cavity perpendicular to the PFH;
(3) plane and parallel to the PFH, passing through the most caudal and lateral ends of the pyriform horizontal plane perpendicular to the mid-sagittal aperture;
(4) two vertical lines perpendicular to the PFH dividing the right and left nasal cavity, and each half was divided into equal thirds named “1, 2, 3” in the latero-medial direction;
(5) to evaluate the bidimensional morphology of the turbinates, the nasal cavities were divided into three thirds proceeding latero-medially. The extension was assessed by analyzing the area of radiopacity delineated by the turbinate in each nasal cavity, considering only the *II* and *III* third sections (Figure 3). The turbinates were considered hypertrophic when the radiopaque area was identified in the mesial third *III.*
(6) the nasal septal deviation was assessed by constructing an angle between the mid-sagittal plane, passing from the Crista galli to the ANS, perpendicular to the floor of the nasal cavity, and a line from the Crista galli to the point of septal convexity, which is located at the outermost point of the projection of the deviated nasal septum, inside the nasal cavity. The severity will be greater according to the deviation that the septum presents in the mid-lateral direction (Figure 4). An angle between 0 and 2° was considered as “No deviation”; between 3° and 5° as “Moderate deviation”, and greater than 5° as “Severe deviation”.

The measurement software used was Blue Sky Plan Version 5.0.25 (Libertyville, IL, USA). The operators were calibrated and supervised by experienced orthodontic clinicians to ensure the accuracy of cephalometric analysis. No more than 10 images were traced in a single day, and repeat analyses were separated by a two-week interval.

Subject’s demographic information and the presence of maxillary constriction were reported in the initial diagnosis of the subjects. The nasal obstruction due to hypertrophy of the turbinates or nasal septal deviation was analyzed, and these parameters were associated with the maxillary constriction.

## 3. Statistical Analysis

To assess the reproducibility of the angular and linear measurements, the Method Error was calculated by remeasuring 25 records randomly selected from the whole sample by a second operator and evaluating any differences in the reported values.

Continuous variables were reported as mean and standard deviation or median and interquartile range (IQR) according to their distribution. Categorical variables (i.e., maxillary constriction, nasal septal deviation, and hypertrophy of the turbinate) were reported as percentages. Interval estimation of the prevalence of the considered variables within the gender groups was performed by the Clopper–Pearson method with a 95% confidence level. Associations between max constriction and the presence of nasal anatomical findings (including turbinate hypertrophy, nasal deviation, and nasal deviation > 5°) were assessed using odds ratios (ORs) with corresponding 95% confidence intervals (95% CIs). For each binary outcome, 2 × 2 contingency tables were constructed by comparing patients with and without max constriction. ORs were calculated to estimate the strength and direction of the association.

## 4. Results

Sample size was determined based on a comparison of two independent proportions (prevalences) by dividing subjects into two groups according to maxillary constriction. The calculation was made assuming a statistical significance level (alpha) of 0.05 (95% Clopper–Pearson confidence level) and a statistical power (1-beta) of 0.80. To ensure a maximum effect size of 0.33 (corresponding to a small-to-medium effect size according to Cohen’s classification), it was necessary to consider a minimum of 40 subjects in each group.

The reproducibility assessment demonstrated high agreement between the original and repeated measurements. The Dahlberg error values were low, reflecting minimal random measurement error, and no statistically significant systematic differences were found between the two sets of measurements (*p* > 0.05). These findings confirm that the measurement method is highly reproducible.

The total sample after the PA collection from the retrospective initial records was composed of 92 subjects, forty-nine males and forty-three females, with a mean age of 13.6 years. After cephalometric assessment, the experimental group with maxillary constriction was composed of 42 subjects, whereas the control group with normal transverse width included 50 subjects. Statistically significant differences were found in maxillary and mandibular widths between the two groups (Table 1).

The prevalence of maxillary constriction in females was 55.81%, while in males it was 36.73% (Table 2).

The data collected on PA was used to assess the relationship between maxillary constriction and the potential anatomical changes associated with nasal obstruction. Patients presenting maxillary constriction showed higher odds of turbinate hypertrophy compared with those without transverse maxillary deficiency, with an estimated odds ratio (OR) of 1.81 (95% CI: 0.76–4.34; *p* = 0.27). This finding suggests that subjects with maxillary constriction had approximately 81% greater odds of exhibiting turbinate hypertrophy than those without maxillary constriction. Nevertheless, the association did not reach statistical significance according to the conventional threshold of *p* < 0.05. The 95% confidence interval was relatively wide and included the null value of 1, indicating uncertainty regarding both the strength and the direction of the true association. Moreover, the *p*-value was well above the threshold for significance, further supporting the absence of a statistically reliable relationship between the two variables. Although the observed odds ratio may suggest a possible tendency toward increased prevalence of turbinate hypertrophy in patients with maxillary constriction, the present data do not provide sufficient evidence to confirm a significant association. Therefore, no definitive relationship between maxillary constriction and turbinate hypertrophy can be established in this sample (Table 3).

The nasal septal deviation was assessed by measuring the previously mentioned angle (Figure 4), and it appeared, regardless of deviation angle and gender, to be very frequent in the analyzed population. Specifically, the subjects were divided into three subgroups based on the angular width of each category, increasing progressively.

Maxillary constriction showed an inverse association with the presence of nasal deviation, with an estimated odds ratio (OR) of 0.34 (95% CI: 0.11–1.06; *p* = 0.06). This finding suggests that subjects presenting maxillary constriction had approximately 66% lower odds of exhibiting nasal deviation compared with those without transverse maxillary deficiency. Although the magnitude of the association appears clinically relevant, statistical significance was not achieved according to the conventional threshold of *p* < 0.05. In particular, the upper bound of the 95% confidence interval slightly exceeded 1, indicating uncertainty regarding the true direction and strength of the association. Therefore, this result should be interpreted as indicative of a borderline trend rather than as evidence of a definitive protective effect of maxillary constriction against nasal deviation (Table 4).

Similarly, when considering more severe nasal deviation, defined as a deviation of the nasal septum greater than 5°, maxillary constriction was again associated with lower odds of the outcome (OR = 0.68; 95% CI: 0.29–1.60; *p* = 0.40). In this case, patients with maxillary constriction showed a 32% reduction in the odds of presenting a nasal deviation > 5° compared to subjects without maxillary constriction. However, the association was weaker and clearly not statistically significant. The wide confidence interval, encompassing values both below and above unity, together with the relatively high *p*-value, indicates substantial variability and lack of evidence for a meaningful relationship between these variables. Consequently, no significant association can be established between maxillary constriction and the presence of severe nasal deviation in the present sample (Table 5).

## 5. Discussion

Postero-anterior cephalograms provide a simplified and accessible evaluation of craniofacial structures; however, they are affected by structural superimposition, magnification errors, and the inability to assess the anteroposterior dimension. The accuracy in identifying anatomical variations, such as turbinate hypertrophy, should be better diagnosed in the 3D evaluations when needed [16].

The following study was conducted with bi-dimensional X-ray analysis of the head in growing subjects with permanent dentition, a conventional diagnostic method to visualize craniofacial structures in the frontal and sagittal planes [17]. In the recent literature, several publications have reported the consequences of maxillary expansion on the nasal cavities, air volume, and reduction in the resistance of airflow passing through the nose. Indeed, the relationship between increased cavity width and nasal permeability has been examined as a potential beneficial effect of rapid maxillary expansion [18,19,20].

Nasal obstruction in children can also result in further significant complications, including otitis media with effusion and sleep disturbances, often associated with the obstructive sleep apnea syndrome (OSAS) [21].

To have quantified volumetric changes in craniofacial structures, diagnostic tools such as CBCT have been used in recent years, which allow a three-dimensional study of structural changes with minimal distortion while emitting a lower radiation dose than conventional CT but still higher than posteroanterior radiographs [13,22,23,24,25,26].

Thus, the relationship between airway size and breathing pattern is a topic of debate in the scientific community. For example, Warren et al. concluded that generally, a larger nasal airway size is also associated with a greater degree of nasal breathing [26]. It has also been observed that as the width of the hard palate increases, there is an increase in the size of the upper airway [27]. This is evident when nasal obstruction is the cause of oral breathing, which in turn affects the transverse dimension of the maxilla [28].

The inferior turbinate, the largest among the three conchae within the nasal cavity, extends perpendicularly along the entire length of the nasal cavity, and among all the anatomical factors analyzed, its hypertrophy significantly decreases the overall volume of the upper airway. The assessment of its anatomy in this study was made by considering its extension in the latero-medial direction on two-dimensional diagnostic images. The turbinates may not show significant enlargement in the anterior portion of the nasal cavity; however, they may be contributing to an obstruction in the region of the posterior choanae. Such an obstruction could potentially play an important role in explaining the discrepancies observed between the outcomes of our study and the findings reported in previous investigations that utilized three-dimensional imaging techniques.

Deviation of the nasal septum is another of the etiologic factors that can result in reduced nasal airflow. Several studies have found a positive effect of maxillary expansion on children’s septal asymmetries through increasing the size and volume of the nasal cavities and thus decreasing airflow resistance and promoting the growth of the entire maxillary complex [29,30]. However, in the present study, a slight association, although not statistically significant, was observed between decreased maxillary transverse dimension and nasal septum deviation. The lack of interdisciplinary consensus between orthodontists and otorhinolaryngologists makes this subject a highly debatable one [31].

Studies performed on CBCT, using an overlapping measurement protocol, have indeed shown a direct association between lower turbinate hypertrophy and the presence of maxillary constriction and a reduction in airflow within the nasal cavities, while no association was found between nasal septal deviation and maxillary transverse dimension. In any case, the definition of turbinate hypertrophy is based on radiographic criteria and is not clinically standardized.

Reflecting the structural superimposition in cephalograms, the potential for measurement bias or misinterpretations should be mentioned in more detail. In addition, a certain degree of septal deviation or enlargement of the turbinate may be included in normal deviation. It would be important to distinguish these differences to evaluate the exact relationship between variables.

Thus, the main limitations of the study lie both in the two-dimensionality of the radiographic images on which the measurements are taken, which do not allow the structures to be evaluated anteroposteriorly, and in the small size of the sample, which may not make associations of clinical relevance evident.

Furthermore, relevant factors such as mouth breathing, allergies, adenoids, body mass index (BMI), and gender were not analyzed in a multivariate model.

The lack of multivariate analysis may have influenced the observed associations. Therefore, it can be concluded that the PA-projected cephalograms are not indicated if exclusively aimed at the evaluation of the nasal cavities due to the limitations of this diagnostic tool for a more thorough evaluation of the entire nasal cavities and their anatomical structures, as well as their impact on nasal flow and volume.

Within these limitations, the strength of this study was to assess, for the first time, a PA analysis of turbinate hypertrophy and nasal septal deviation, considering the abovementioned ALARA principle to be followed in most countries for children and adolescents, which does not justify a 3D evaluation in growing patients, only in cases of nasal obstruction.

Hence, within the framework of orthodontic diagnosis in growing patients and in accordance with the ALARA principle, postero-anterior (PA) cephalograms should not be considered a definitive diagnostic tool for nasal obstruction but rather a preliminary screening method. Their routine use in orthodontic records allows clinicians to identify potential anatomical variations, such as turbinate hypertrophy or nasal septal deviation, that may be associated with impaired nasal airflow. In this context, PA imaging provides an accessible and low-radiation approach to detect early signs that may warrant further multidisciplinary evaluation.

An expansion of the sample size, possibly with multicenter studies, might be useful to reconsider our results, eventually applying artificial intelligence (AI)-driven automated PA cephalometric analysis for future investigations [31].

Moreover, it should be considered that nasal morphology is not a static feature but changes with age and growth, showing a dynamic interaction with overall craniofacial development. In particular, nasal dimensions and shape are closely correlated with craniofacial morphology, as changes in maxillary and mandibular growth patterns may influence nasal architecture and vice versa [32]. Additionally, congenital craniofacial conditions may significantly affect nasal morphology and should be considered as potential confounding factors in the interpretation of results. In particular, cleft lip and/or palate, Binder syndrome, Goldenhar syndrome, Treacher Collins syndrome, and craniosynostoses are known to be associated with altered nasal anatomy and maxillofacial growth patterns, which may influence both nasal airway dimensions and craniofacial relationships in affected individuals [33].

## 6. Conclusions

The initial hypothesis was not supported, as no statistically significant association was found between lower turbinate hypertrophy, nasal septal deviation, and a reduction in maxillary transverse skeletal dimensions assessed on postero-anterior cephalograms. However, a deviated nasal septum could be slightly associated with a maxillary constriction. A higher percentage of females showed maxillary constriction, but with no significant differences due to gender in our sample.

Present findings should be interpreted with caution due to the inherent limitations of two-dimensional imaging. Future studies with three-dimensional evaluations in a wider sample could provide further outcomes to be discussed.

## Figures and Tables

**Table 1:** Comparison of the starting group between groups (experimental vs. control).

	Group		
Characteristic	Experimental, *N* = 42 ^1^	Control, *N* = 50 ^1^	*p*-Value ^2^
Age	14.75 (13.00, 16.00)	13.40 (12.00, 14.60)	0.01
Maxillary width	60.00 (59.00, 63.00)	67.00 (65.25, 68.00)	<0.001
Mandibular width	81.00 (80.00, 84.00)	85.00 (83.00, 86.00)	<0.001
Difference	21.00 (19.25, 23.00)	18.00 (17.00, 19.00)	<0.001
Ratio	75.00 (72.31, 76.24)	79.06 (78.16, 79.70)	<0.001

^1^ Median (Q1, Q3) or Frequency (%); ^2^ Wilcoxon rank sum test.

**Table 2:** Estimated prevalence of maxillary constriction within gender groups. 95% Clopper–Pearson confidence intervals.

Gender	Total Subjects	N° with Maxillary Constriction	Prevalence	Low	Upper
Female	43	24	55.81	39.88	70.92
Male	49	18	36.73	23.42	51.71

**Table 3:** Association table of maxillary constriction and turbinate hypertrophy.

		Turbinate Hypertrophy		
		No	Yes	Total
**Max Constriction**	No	21	29	50
	Yes	12	30	42
	Total	33	59	92
	**OR = 1.81, 95% CI: 0.76–4.34**			

**Table 4:** Association table of nasal septal deviation and maxillary constriction.

		Nasal Deviation		
		No	Yes	Total
**Max Constriction**	No	6	44	50
	Yes	12	30	42
	Total	18	74	92
	**OR = 0.34, 95% CI: 0.11–1.06**			

**Table 5:** Association table of maxillary constriction and nasal deviation higher than 5 degrees.

		Nasal Deviation > 5°		
		No	Yes	Total
**Max Constriction**	No	25	25	50
	Yes	25	17	42
	Total	50	42	92
	**OR = 0.68, 95% CI: 0.29–1.60**			

## Data Availability

The original contributions presented in this study are included in the article/Supplementary Material. Further inquiries can be directed to the corresponding author.

## Supplementary Materials

The following supporting information can be downloaded at: https://www.mdpi.com/article/10.3390/children13050673/s1, Table S1: Values of maxilla-mandibular discrepancy in the experimental with maxillary constriction (a), and the control group with normal values (b).
